# TGF-β pathway activation by idiopathic pulmonary fibrosis (IPF) fibroblast derived soluble factors is mediated by IL-6 trans-signaling

**DOI:** 10.1186/s12931-020-1319-0

**Published:** 2020-02-18

**Authors:** Gali Epstein Shochet, Elizabetha Brook, Becky Bardenstein-Wald, David Shitrit

**Affiliations:** 1 Pulmonary Medicine Department, Meir Medical Department, 59 Tchernichovsky St, 44281 Kfar Saba, Israel; 2 Sackler Faculty of Medicine, Tel Aviv University, Tel Aviv, Israel

**Keywords:** IL-6, Fibroblasts, STAT3, Fibrosis, Tocilizumab, sIL-6R, Gremlin (GREM1), Smad3

## Abstract

**Background:**

Idiopathic pulmonary fibrosis (IPF) is a chronic and ultimately fatal disease characterized by a progressive decline in lung function. Fibrotic diseases, such as IPF, are characterized by uncontrolled activation of fibroblasts. Since the microenvironment is known to affect cell behavior, activated fibroblasts can in turn activate healthy neighboring cells. Thus, we investigated IPF paracrine signaling in human lung fibroblasts (HLFs) derived from patients with IPF.

**Methods:**

Primary human fibroblast cultures from IPF (IPF-HLF) and control donor (N-HLF) lung tissues were established and their supernatants were collected. These supernatants were then added to N-HLFs for further culture. Protein and RNA were extracted from IPF/ N-HLFs at baseline. Interleukin-6 (IL-6) and TGF-β-related signaling factors (e.g. STAT3, Smad3) were evaluated by western blot and qPCR. IL-6 levels were measured by ELISA. IL-6 signaling was blocked by Tocilizumab (TCZ) (10 ng/ml).

**Results:**

IPF-HLFs were found to significantly overexpress IL-6 receptor (IL-6R), suppressor of cytokine signaling 3 (SOCS3), phospho-STAT3-Y705 and phospho-Smad3 in comparison to N-HLFs (*p* < 0.05). In addition, they were found to proliferate faster, secrete more IL-6 and express higher levels of the soluble IL-6R. IPF-HLF increased proliferation was inhibited by TCZ. Moreover, IPF-HLF derived supernatants induced both direct and indirect STAT3 activation that resulted in Smad3 phosphorylation and elevated Gremlin levels in N-HLFs. These effects were also successfully blocked by TCZ.

**Conclusions:**

IPF-HLF paracrine signaling leads to IL-6R overexpression, which in turn, affects N-HLF survival. The IL-6/STAT3/Smad3 axis facilitates cellular responses that could potentially promote fibrotic disease. This interplay was successfully blocked by TCZ.

## Background

Idiopathic pulmonary fibrosis (IPF) is a chronic and progressive lung disease associated with significant morbidity and poor prognosis [1]. Fibroblasts exhibit phenotypic divergence within the normal lung, while this heterogeneity was shown to be significantly greater in the IPF lung [2, 3]. Activated fibroblasts were shown to be the key components in the fibrotic process. Their interaction with the microenvironment, especially the immune cells, was shown to contribute to the disease progression. In our previous studies, we already showed that the fibroblasts can secrete pro-fibrotic and pro-angiogenic signals that promote disease progression [4].

Fibrosis, in response to tissue damage or persistent inflammation, is a pathological hallmark of many chronic degenerative diseases [1, 5]. If injury persists, the wound healing process passes through an inflammatory phase, with increased levels of interleukin-1 (IL-1) and tumor necrosis factor-alpha (TNF-α), leading to tissue remodeling. Interleukin-6 (IL-6) is another proinflammatory cytokine, which is produced by a wide variety of cells, including fibroblasts [3]. IL-6 was shown to be elevated in lungs of IPF patients [6] and in mouse models of pulmonary fibrosis [7]. Moreover, IL-6-deficient (IL-6(−/−)) mice had relatively attenuated fibrotic changes following bleomycin treatment in comparison to the wild type controls [8]. Recent studies showed that the IL-6 can also promote fibrosis by driving chronic inflammation [5] and by activating the TGFβ pathway [9, 10], which is the most potent profibrotic cytokine known [11–13].

The IL-6 receptor (IL-6R), usually membrane-bound, can also exist in a soluble form (sIL-6R). In this form, IL-6 binds to sIL-6R, resulting in a complex that activates the membrane bound glycoprotein 130 (gp130), which is constitutively expressed on most cell types. This process also results in Jak/signal transducer and activator of transcription (STAT) signaling pathway activation [14] and is termed IL-6 trans-signaling [15]. This signaling pathway was already implicated in a variety of inflammatory processes, including rheumatoid arthritis (RA) [16], systemic sclerosis (SS) [17], cancer [18], as well as IPF [19, 20]. Importantly, unlike other soluble cytokine receptors, sIL-6R does not act antagonistically by limiting the IL-6 cytokine activity, but rather agonistically. The sIL-6R is formed either by limited proteolysis of membrane bound receptors or directly secreted from the cells following alternative mRNA splicing [8, 9].

Tocilizumab (TCZ), an anti-human IL-6R neutralizing antibody, which prevents binding of IL-6 to IL-6R, thus inhibiting both classic and trans-signaling pathways, is approved for the treatment of RA [21]. This drug was also implicated in other inflammatory conditions that involve a fibrotic phenotype, such as SS [17]. Results from the phase II randomized controlled trial (faSScinate) of SS patients showed forced vital capacity (FVC) stabilization within the TCZ receiving SS patients in comparison to placebo controls [22], as well as in placebo-treated patients who later transitioned to TCZ in the open label period [23].

Moreover, the SENSCIS™ trial for SS associated interstitial lung disease (SS-ILD) showed that the treatment that is already proven effective for IPF (i.e. nintedanib) could also be effective for SS-ILD [24]. We hypothesized that it could also be vice versa. Thus, we studied IL-6 related signaling in primary human lung fibroblasts (HLFs) taken from patients with IPF. We used our established IPF supernatants (IPF-SN) model to examine whether IPF-HLFs secrete factors that activate IL-6 signaling, as well as the impact of TCZ on this process.

## Methods

### Lung fibroblast culture

Primary human lung fibroblasts (HLF) were isolated as previously described [25], from lung tissue samples from histologically confirmed patients with IPF and normal control donors. Following extraction, the fibroblasts were cultured in Dulbecco’s modified eagle’s medium (DMEM) supplemented with 15% fetal calf serum (FCS), L-glutamine (2 mM), and Pen-Strep-Nystatin antibiotics (Biological Industries, Israel). Cells were maintained in 5% CO_2_ at 37 °C. The culture media were collected from the confluent culture flasks and stored at − 80 °C. Media was collected up to passage 8 and as long as the cells proliferated normally.

### Exposing lung fibroblasts to supernatants (SN)

Fibroblasts (2 × 10^5^) were seeded in 24-well plates and allowed to adhere for 24 h prior to the beginning of experiments. Then, IPF-HLF or N-HLF-derived SN (400 μl) was added for further culture. TCZ (anti-IL-6R) (10 ng/ml, Actemra®, Roche) was dissolved in PBS.

### Cell count

Cell number and viability were evaluated by the max quant analyzer (Miltenyi Biotec, Bergisch Gladbach, Germany). Results were verified by manual counting of Trypan blue-stained cells.

### Western blotting

N-HLF, IPF-HLF or 10 mg of lung tissue samples were lysed, and Western blot was performed as previously described [26]. For phospho/total (p/t) Smad3, p/t Stat3 and suppressors of cytokine signaling 3 (SOCS3) expression levels, proteins were extracted directly from the tissue sample (10 mg, following biopsy) and from the cell line culture flasks during regular cell passages (between passages 3–10, during the normal proliferation phase). The following rabbit/mouse anti-human antibodies were used: phospho-Stat3 Tyr705 (#9145), phospho-Stat3 S727 (#9134), Stat3 (#9139), phospho-Smad3 (#9520) and Smad3 (#9523) from Cell Signaling Technologies, USA; Glyceraldehyde-3-Phosphate Dehydrogenase (GAPDH) (ab9484) and SOCS3 (ab16030) were purchased from Abcam, USA; alpha-Tubulin (T5168) was purchased from Sigma, USA. Bound antibodies were visualized using Goat peroxidase-conjugated secondary antibodies (Millipore, USA, anti-Mouse IgG #AP308P and anti-Rabbit IgG #AP307P) followed by enhanced chemiluminescence detection (Millipore, USA). Results were normalized to Tubulin and GAPDH.

### Search tool for the retrieval of interacting genes/proteins (STRING) analysis

PPI networks were constructed for the protein products using information from the Search Tool for the Retrieval of Interacting Genes/Proteins (STRING, v11; http://string-db.org/) [27].

### RNA extraction and RT cDNA synthesis

RNA was extracted using the RNeasy kit (QAIGEN, USA). Extracted RNA was converted to cDNA using GeneAmp (Applied Biosystems, USA).

### Real time quantitative PCR

Reactions were done using Power SYBR Green (Applied Biosystems, UK). Primers sequences (purchased from Hylabs, Israel) are listed in Table 1. Primers were normalized by specific cDNA standard curves obtained from known amounts of cDNA. GAPDH served as the housekeeping control.

### ELISA based antibody array

IL-6 levels were measured using Quantibody Human ANG-Q1 kit (RayBiotec, Inc., USA), according to the manufacturer’s instructions.

### Statistical analysis

Statistical analysis was done using GraphPad Prism version 7.00 for Windows (GraphPad Software, La Jolla California USA, www.graphpad.com). ANOVA was performed to compare differences between multiple cohorts. Paired Student’s t-tests were employed to analyze differences between two groups. An effect was considered significant when the *P*-value was < 0.05. All experiments were repeated at least three times.

## Results

### IPF-HLFs secrete high levels of IL-6 and activate the STAT3 pathway in normal HLFs

**Fig. 1:**
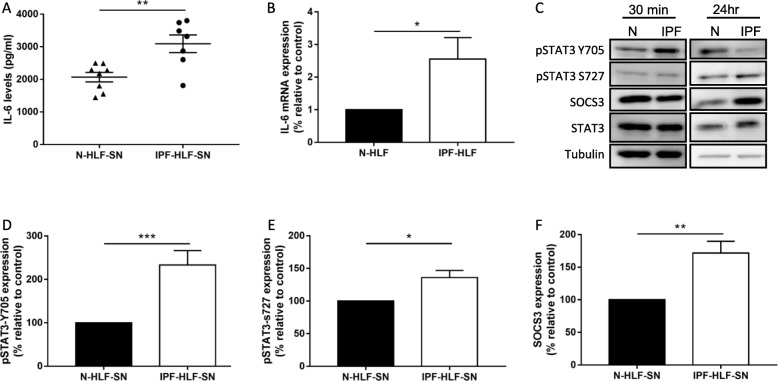
*IPF-HLFs secrete high levels of IL-6 and activate the STAT3 pathway in normal HLFs.* Human lung fibroblasts from patients with IPF (IPF-HLF) or control donors (N-HLF) and were cultured and their supernatants (SN) were collected. IL-6 levels in the SN were measured by ELISA-based array **a**. IL-6 mRNA levels from N-HLF and IPF-HLF cells were measured by qPCR **b** SN from IPF-HLFs was added to N-HLF for further cultures. The effect of the IPF-HLF-SN on N-HLF pSTAT3-Y705 (30 min, **c-d**, pSTAT3-S727 (24 **h, e** and total protein levels of SOCS3 (24 **h, f** were analyzed by western blotting. **c** Representative western blots for Figs. D-F. *p < 0.05, **p < 0.01, and ***p < 0.001. (*n* ≥ 4).

The microenvironment significantly affects disease progression [28]. In our previous works, we showed that IPF paracrine signaling significantly alters N-HLF phenotype [4]. Most works discussing IL-6 usually suggest that it is secreted from neighboring immune cells (e.g. macrophages). Here, we measured IL-6 levels in the IPF-HLF derived supernatant (IPF-HLF-SN) and tested whether the IPF-HLF-SN could activate the STAT3 pathway. In fact, the IPF-HLF-SN was found to contain high levels of IL-6 in comparison to N-HLF-SN (Fig. 1a). In addition, the expression level of IL-6 mRNA was also significantly higher in the IPF-HLFs in comparison to N-HLFs at baseline (Fig. 1b). Direct STAT3 activation was observed following 30 min of N-HLF exposure to the IPF-HLF-SN (Fig. 1c-d). Following 24 h, there was also an increase in pSTAT3-S727 and SOCS3 levels (Fig. 1e-f).

**Table 1:** List of primers

	Forward (5′-3′)	Reverse (5′-3′)
IL-6	GGTACATCCTCGACGGCATCT	GTGCCTCTTTGCTGCTTTCAC
IL-6R	GCTGTGCTCTTGGTGAGGAAGTTT	CTGAGCTCAAACCGTAGTCTGTAGAAA
sIL-6R	GCGACAAGCCTCCCAGGTT	CCGCAGCTTCCACGTCTTCTT
GREM1	TATGAGCCGCACAGCCTACA	GCACCTTGGGACCCTTTCTT
ACTA2	TGAGAAGAGTTACGAGTTGCCTGAT	GCAGACTCCATCCCGATGAA
COL1a	CGAAGACATCCCACCAATCAC	CAGATCACGTCATCGCACAAC
TGFB1	TTTTGATGTCACCGGAGTTG	AACCCGTTGATGTCCACTTG
GAPDH	CTCTGCTCCTCCTGTTCGAC	TTAAAAGCAGCCCTGGTGAC

### The IL-6 pathway is overexpressed in IPF-HLFs

**Fig. 2:**
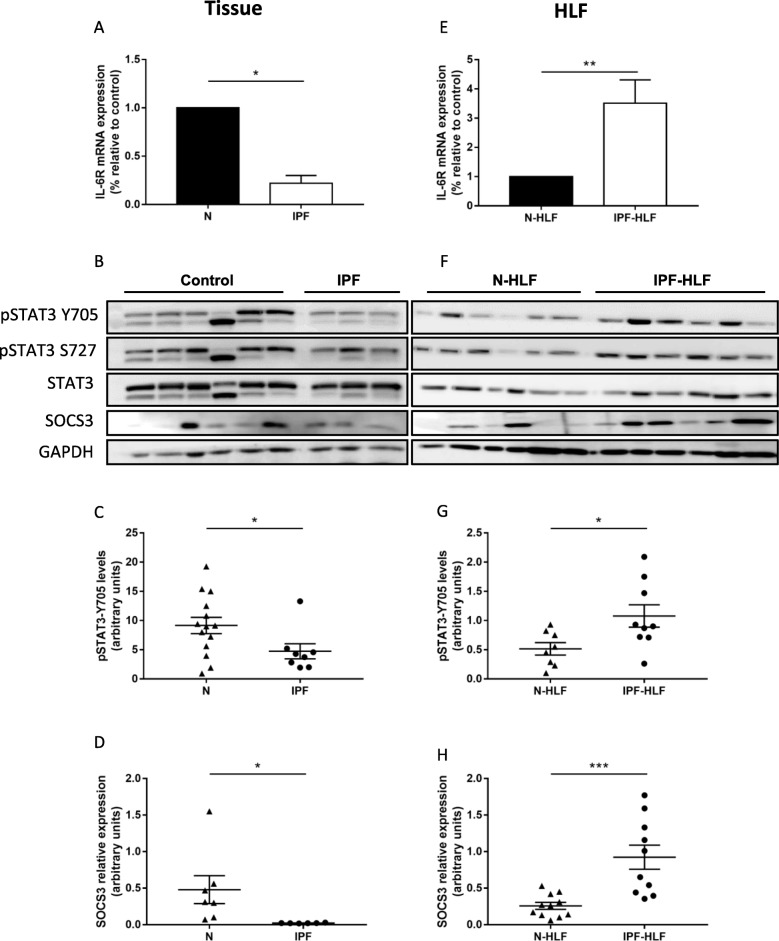
*The IL-6 pathway is overexpressed in IPF-HLFs*. RNA and proteins were extracted from IPF (*n* = 8) and control donor lung tissue lysates (*n* = 8) **a-d**, as well as from human lung fibroblasts (HLF) derived from patients with IPF (*n* = 10) or control donors (*n* = 14) (N) **e-h**. IL-6R mRNA was measured by qPCR **a,e**. pSTAT3-Y705 **c,g** and SOCS3 protein (D,H) levels were measured by western blotting. **b,f** Representative western blots for Figs. **c-d** and **g-h**. *p < 0.05, **p < 0.01, and ***p < 0.001 (*n* ≥ 4)

In order to test the relevance of the above findings, IPF patient and control donor lung samples were tested for IL-6R, pSTAT3 and SOCS3 levels. Patient baseline characteristics are shown in Table 2. Interestingly, IL-6R, as well as pSTAT3 levels were significantly lower in the IPF tissue samples (Fig. 2a-c). SOCS3 expression in IPF tissues was also reduced (Fig. 2d). However, when analyzing IPF-HLFs in comparison to N-HLFs, we found a significant overexpression of IL-6R (Fig. 2e). In addition, similarly to the effects of the IPF-HLF-SN, there was an elevated baseline expression of pSTAT3-Y705 and SOCS3 in these cells (Fig. 2f-h). These findings show that the STAT3 pathway is excessively activated in IPF-HLFs at baseline.

**Table 2:** Lung sample patient characteristics

	Control donors *N* = 19	IPF *N* = 10	*P*-Value
Age	66.4 ± 15	58 ± 9	0.08
Gender (% male)	58%	70%	0.7
Smoker (%)	42%	16%	0.41
FVC %	99.8 ± 17	64.3 ± 15	< 0.001
DLCO %	74.3 ± 12	48.7 ± 4.4	0.001

### Phosphorylated and total Smad3 are overexpressed in IPF-HLFs

**Fig. 3:**
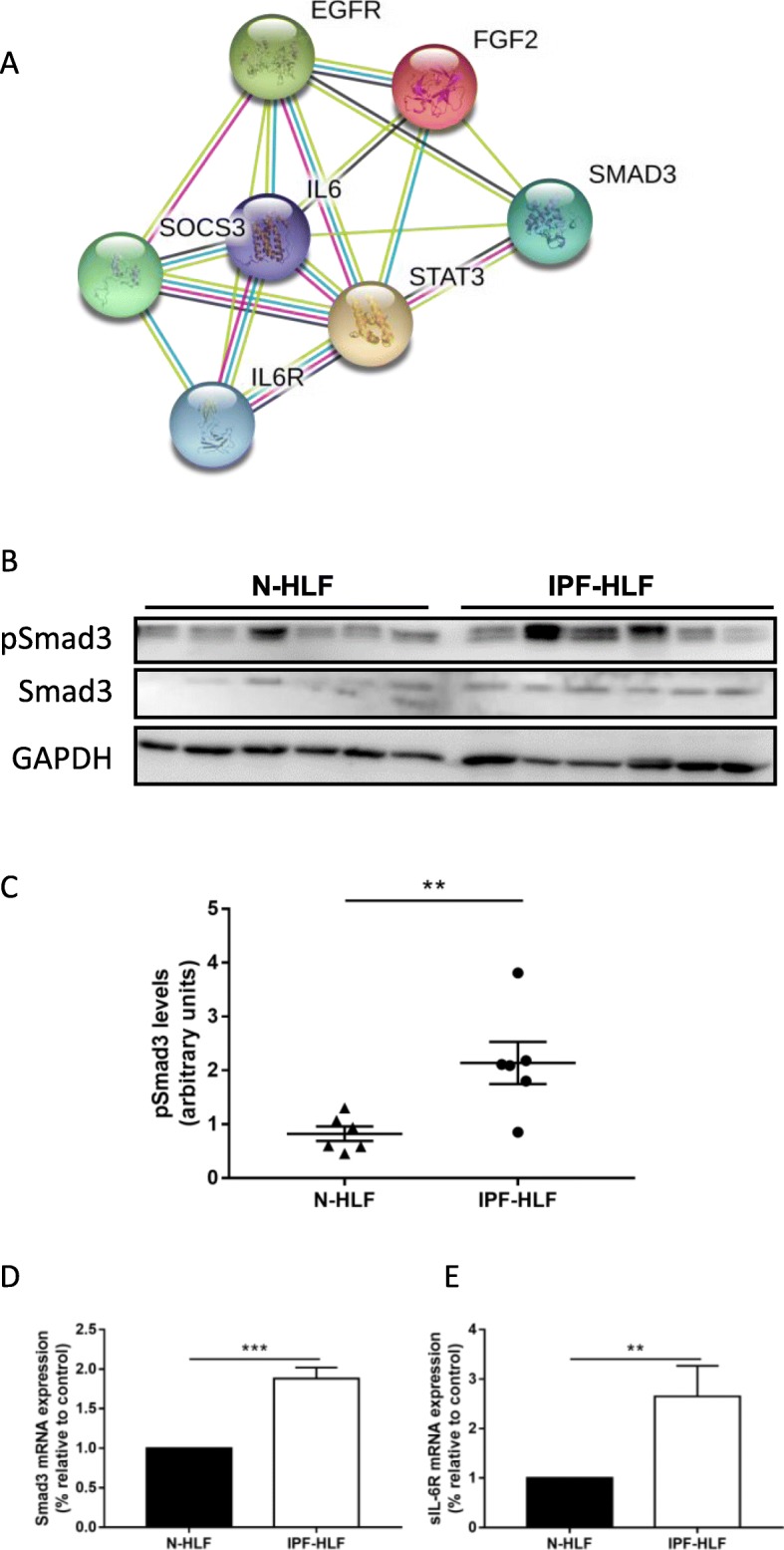
*Phosphorylated and total Smad3 are overexpressed in IPF-HLFs.* Protein interaction networks were constructed using STRING (http://string-db.org/) **a**. Proteins and RNA were extracted from human lung fibroblasts from patients with IPF (IPF-HLF) or control donors (N-HLF). phospho and total Smad3 protein levels were tested using Western Blot **b-c**. Smad3 **d** and soluble IL-6R **e** mRNA levels were measured by qPCR. *p < 0.05, **p < 0.01, and ***p < 0.001 (*n* ≥ 4)

Transforming growth factor-β (TGF-β) is another well discussed pathway in fibrosis development [19]. Recent publications [9, 29, 30], suggested Smad3 as a possible target of the IL-6 trans-signaling. Therefore, STRING analysis was performed, with the inclusion of the aforementioned targets, as well as Smad3. In addition, we added Epidermal growth factor receptor (EGFR) and FGF-2 that were recently discovered by our group as elevated in IPF-HLFs [4, 27]. Indeed, Smad3 was found to be significantly linked in this protein interaction network (Fig. 3a). Moreover, we found significantly higher levels of phosphorylated Smad3, as well as Smad3 mRNA in IPF-HLFs in comparison to N-HLF cells (Fig. 3b-d). Supporting the hypothesis of the IL-6 trans-signaling, the soluble form of IL-6R (sIL-6R) was also elevated in these cells (Fig. 3e).

### IPF-HLF derived soluble factors activate pSmad3 via IL-6 trans-signaling in normal HLFs

**Fig. 4:**
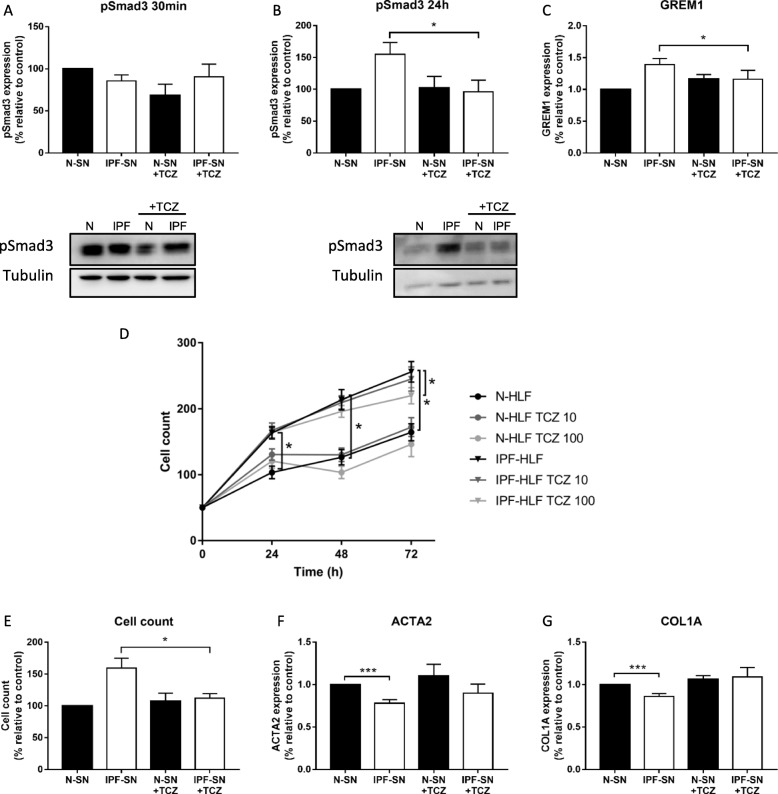
*IPF secreted factors activate Smad3 and induce cell proliferation* via *IL-6 trans-signaling.* Supernatants (SN) from cultured human lung fibroblasts from patients with IPF (IPF-HLF-SN) were added to lung fibroblasts from control donors without IPF (N-HLF). Effects of the IPF-HLF-SN with/ without Tocilizumab (TCZ, 10 ng/ml) on pSmad3 protein levels and GREM1 mRNA levels were tested by Western Blot **a-b** and qPCR **c**, respectively. Lung fibroblasts derived from patients with IPF (IPF-HLF) or from control donors (N-HLF) were cultured with/ without TCZ, 10 and 100 ng/ml. Cell growth was monitored at 24, 48, and 72 h. At 24 h, culture media was changed and TCZ was added. Values are means ± SE **d**. One-way ANOVA was used for each time point, with the main effect of IPF-HLF versus N-HLF. *p ≤ 0.05, *n* = 5. The effect of IPF-HLF-SN with/ without TCZ (10 ng/ml) on cell number was tested at 24 h **e**. The effect of IPF-HLF-SN with/ without TCZ (10 ng/ml) on mRNA levels of αSMA (ACTA2) and Collagen1a (COL1A) were tested by qPCR at 24 h (**f-g**). ***p < 0.001

Next, we exposed N-HLFs to IPF-HLF-SN and tested whether pSmad3 is activated. Supporting the assumption of indirect activation (i.e. IL-6 trans-signaling), Smad3 phosphorylation was observed following 24 h and not at 30 min, which is considered the period of direct activation (Fig. 4a-b). Furthermore, there was a significant elevation in Gremlin (GREM1) (Fig. 4c), which was previously shown to be elevated as a result of the IL-6 trans-activation through STAT3 and Smad3 in fibrosis [9].

To test whether it was IL-6 mediated, TCZ that inhibits the IL-6 in both the canonical and the trans-signaling pathways was added to IPF and N-HLF-SNs. In fact, TCZ successfully blocked the elevation in pSmad3 by IPF-HLF-SN at 24 h (*p* < 0.05, Fig. 4b). Accordingly, the elevation in GREM1 was also successfully blocked by TCZ (*p* < 0.05, Fig. 4c). Thus, IPF-HLFs show activated baseline Smad3 phosphorylation, which can be potentially induced by the IPF secreted factors via the IL-6 trans-signaling.

### IL-6 pathway blockage inhibits cell proliferation and affects differentiation

In order to evaluate the importance of IL-6 for the IPF-HLF cell survival, we cultured IPF-HLFs and N-HLFs with TCZ for up to 72 h, and followed their growth daily. As expected, IPF-HLFs proliferated faster than N-HLF (*p* < 0.05, Fig. 4d). In addition, IPF-HLF cell growth was significantly inhibited by TCZ (*p* < 0.05, Fig. 4d), while N-HLFs were not affected. These results suggest that the elevated baseline level of IL-6/ Smad3 in IPF-HLFs is at least in part responsible for the increased proliferation of these cells.

Previously, we showed that IPF-HLF-SN reduces the alpha-smooth muscle actin (α-SMA) and Collagen1a levels in N-HLFs [25]. This was the result of the increased proliferation, and therefore reduced differentiation. Thus, N-HLFs were cultured with N or IPF-HLF-SNs with/ without TCZ for 24 h. Following culture, cells were counted. In support of our previous findings, TCZ prevented the elevation in N-HLF cell counts by the IPF-HLF-SN (p < 0.05, Fig. 4e). In addition, the drug prevented the down-regulation in α-SMA and Collagen1a that were previously observed (*p* < 0.05, Fig. 4f-g). These findings highlight the importance of this pathway in disease progression, since its blockage attenuates fibrotic phenotype.

## Discussion

Fibrotic diseases, such as IPF and SS, are characterized by uncontrolled activation of fibroblasts. This activation was shown to be caused by increased inflammatory cytokines, (e.g. TNFα, IFNγ and IL-6) which are usually considered to be secreted by inflammatory cells (e.g. macrophages). In this study, we showed that IPF-HLFs secrete IL-6, activate the IL-6/ STAT3 and sequentially TGF-β signaling pathways in normal HLF cells in a paracrine manner. This autonomous activation of fibroblasts is thought to mediate progression of fibrosis in later stages of the disease.

Similar results were shown in a recent study by Denton et al. Their work elegantly utilized patient samples from the faSScinate phase II trial in various molecular analyses in dermal fibroblasts, which linked IL-6 to key profibrotic pathways such as the TGF- β [31]. A recent study by Milara et al. showed that IL-6, via STAT3 phosphorylation, induced proliferation and migration of primary human fibroblasts. They also showed that IL-6 is elevated in BAL and in lung tissues of rats following bleomycin treatment [32].

Activation of mesenchymal cells following injury and inflammation results in elevated TGF-β levels and increased cell proliferation, as well as elevated production of various cytokines [33]. Here, we showed that IL-6 secretion is elevated in IPF-HLFs, in addition to elevated IL-6 mRNA levels. Compared with IL-6 classic signaling, trans-signaling shows a different spectrum of IL-6-mediated actions, which is mainly involved in inflammatory diseases and cancer progression [15, 34, 35]. An extensive work by Le et al. [20] showed the importance of the IL-6 trans-signaling in IPF progression. In their work, they suggest that sIL6R is originated from macrophages that in turn, promote the fibrotic process by activating fibroblasts. They also suggest the shedding process of IL-6R by ADAM17 as the source of sIL-6R. Our results support their findings and highlight the involvement of fibroblasts in this process. However, we also show that fibroblast paracrine signaling by itself can initiate such signaling, as the IPF-HLFs express higher mRNA levels of the sIL6R.

The canonical activation of STAT3 relies on the Y705 residue, which results in nuclear translocation and activation of target genes. The STAT proteins translocate to the nucleus to induce the transcription of targets, such as SOCS [36, 37]. However, it is now clear that there are (many) other non-canonical pathways, in which STAT3 can transduce alternative signaling [38, 39]. In our IPF supernatant system, we showed that pSTAT3-Y705 was briefly activated and then down-regulated, giving a rise to pSTAT3-S727 and SOCS3 at 24 h. A similar observation was described by Li et al. that showed an interchange in pSTAT3 residues in Crohn’s disease [33].

In the lungs of patients with IPF, there was no overexpression of IL-6R or elevated levels of pSTAT3-Y705. The level of SOCS3 was also reduced in the IPF derived lungs. This reduction in SOCS3 was previously shown in other fibrotic conditions [33, 40]. Nevertheless, a recent study by Milara et al. showed that lungs from patients with IPF expressed higher levels of STAT3 and JAK2, as well as phosphorylated STAT3 [32]. In another article they showed that this was also the case in pulmonary arteries in IPF [29]. As STAT3 possesses two phosphorylation sites that are known to be relevant to function: pSTAT3-Y705 and pSTAT3-S727 [37], it is important to distinguish between the two. However, they and others [19, 30, 41] didn’t state which of the two phosphorylated STAT3s was tested. Pechkovsky et al. also studied the localization of pSTAT3-Y705 in UIP lungs. They showed localization of the pSTAT3-Y705 mainly in areas of dense fibrosis [2]. Similar findings were shown by O’Donoghue et al. [19] and Pedroza et al. [30]. A possible explanation could be attributed to the fact that our control samples were derived from lobectomies of cancer patients. As STAT3 signaling is activated in cancer, it is possible that although the sections were distant from the tumor and defined ‘normal’ by histology, several molecular pathways were still activated.

Interestingly, they also showed that IPF LFs express high basal levels of pSTAT3-Y705 and FN1 in vitro [2]. When we tested basal levels in HLFs, we also found that IPF-HLFs expressed higher levels of pSTAT3-Y705, as well as IL-6R and SOCS3, in spite of being cultured in-vitro for several passages. The ability of these cells to preserve their fibrotic phenotype was already shown by us and others [2, 3, 25, 41]. Furthermore, SOCS3 expression was shown to be elevated for up to 30 days in bleomycin induced fibrosis [19].

TGFβ is extensively involved in the development of fibrosis in different organs [13, 31, 42], with the interplay between STAT3 and TGF-β pathways is widely discussed. For instance, targeting of JAK-2 in SSc fibroblasts abrogated the pathologic activation of the TGFβ signaling and prevented myofibroblast differentiation [41]. Other studies, such as O’Reilly et al., suggest the IL-6 trans-signaling, as the driver for STAT3 dependent TGF-β pathway activation [9]. In their work, they suggested the Gremlin protein mediates Smad3 activation by the IL-6/STAT3 pathway. As Gremlin was already found to be overexpressed in IPF [43, 44], we tested its expression in our system and found it to be significantly elevated in normal HLFs following exposure to IPF-HLF-SN. It was also significantly overexpressed at the baseline level in IPF-HLFs in comparison to the N-HLFs. Interestingly, in a large recent study by McDonough et al. that characterized the transcriptional regulatory model of fibrosis in the human lung, the GREM1 was found to be one of the four upregulated genes in IPF that also correlated with disease severity [45].

In that work, they also showed that activation of the IL-6 trans-signaling led to an elevation in COL1A, but not in TGF-β1. In our experimental system, we didn’t see such elevation. This could be explained by the fact that they used 20 ng/ml of IL-6, while our IPF-HLF-SN only contained about 2 ng/ml. Nevertheless, at this concentration pSmad3 and GREM1 were significantly elevated. Since this activation was observed only following 24 h, we assumed the activation of pSmad3 was not direct. Moreover, it was successfully inhibited by TCZ indicating IL-6 involvement. Similarly to our results, Pechkovsky et al. also showed that activation of HLF cells with IL-6 resulted in SOCS3 elevation, but did not result in an increase in COL1A levels [2].

TCZ prevents binding of IL-6 to IL-6R thereby inhibiting both classic and trans-signaling pathways [21, 46]. In our experimental system, we showed that IPF-HLFs proliferate faster than N-HLFs at baseline and that the TCZ inhibited this observation. A similar observation was presented by Le et al. [20] showing that baseline proliferation rates of IPF fibroblast cell lines (LL97A) are higher than those of normal fibroblasts (CCD8Lu). Nevertheless, Alvarez et al. reported the opposite, while suggesting a senescent phenotype of IPF derived vs. controls [47]. This difference could be explained by variations in the level of cell differentiation.

As suggested a while ago by Raghu et al., cells cultured from specimens with early fibrosis have a greater proliferative potential than those from late fibrosis [48]. Since Alvarez et al. took only lower lobe specimens, which are known to be the most affected, it is possible that the cells they extracted were more differentiated (and thus less proliferative). In addition, they used enzymatic digestion for cell extraction, while we used the explant culture method [49], which possibly favors the more proliferative fibroblast type.

## Conclusions

IPF-HLFs express high baseline levels of both canonical and IL-6 trans-signaling components, leading to indirect TGF-β pathway activation and potentially to disease progression. Since the treatment for IPF (i.e. nintedanib) is now extended to other ILDs, as well as to SS-ILD, it is possible to assume that treatments for SS could prove effective in IPF. Further studies are needed in order to elucidate the benefit of this treatment in ILD patients.

## Data Availability

I do not have a cite that I can upload the files into. Any data can be supplemented on demand.

## Abbreviations

EGFR: Epidermal growth factor receptor
FVC: Forced vital capacity
GAPDH: Glyceraldehyde-3-Phosphate Dehydrogenase
HLF: Human lung fibroblasts
IL-6: Interleukin-6
IL-6R: Interleukin-6 receptor
ILD: Interstitial lung disease
IPF: Idiopathic pulmonary fibrosis
N-HLFs: Normal human lung fibroblasts
p: Phospho
RA: Rheumatoid arthritis
sIL-6R: Soluble interleukin-6 receptor
SN: Supernatant
SOCS3: Suppressors of cytokine signaling 3
SS: Systemic sclerosis
STAT3: Signal transducer and activator of transcription
STRING: Search Tool for the Retrieval of Interacting Genes/Proteins
TCZ: Tocilizumab
TGF-β: Transforming growth factor-β
α-SMA: Alpha-smooth muscle actin

## References

[1] Sgalla G, Iovene B, Calvello M, Ori M, Varone F, Richeldi L (2018). Idiopathic pulmonary fibrosis: pathogenesis and management. Respir Res.

[2] Pechkovsky DV, Prele CM, Wong J, Hogaboam CM, McAnulty RJ, Laurent GJ, Zhang SS, Selman M, Mutsaers SE, Knight DA (2012). STAT3-mediated signaling dysregulates lung fibroblast-myofibroblast activation and differentiation in UIP/IPF. Am J Pathol.

[3] Larsson O, Diebold D, Fan D, Peterson M, Nho RS, Bitterman PB (2008). Henke CA fibrotic myofibroblasts manifest genome-wide derangements of translational control. PLoS One.

[4] Epstein Shochet Gali, Brook Elizabetha, Eyal Omer, Edelstein Evgeny, Shitrit David (2019). Epidermal growth factor receptor paracrine upregulation in idiopathic pulmonary fibrosis fibroblasts is blocked by nintedanib. American Journal of Physiology-Lung Cellular and Molecular Physiology.

[5] Fielding CA, Jones GW, McLoughlin RM, McLeod L, Hammond VJ, Uceda J, Williams AS, Lambie M, Foster TL, Liao CT (2014). Interleukin-6 signaling drives fibrosis in unresolved inflammation. Immunity.

[6] Zhou Y, Murthy JN, Zeng D, Belardinelli L, Blackburn MR (2010). Alterations in adenosine metabolism and signaling in patients with chronic obstructive pulmonary disease and idiopathic pulmonary fibrosis. PLoS One.

[7] Pedroza M, Schneider DJ, Karmouty-Quintana H, Coote J, Shaw S, Corrigan R, Molina JG, Alcorn JL, Galas D, Gelinas R (2011). Blackburn MR Interleukin-6 contributes to inflammation and remodeling in a model of adenosine mediated lung injury. PLoS One.

[8] Saito F, Tasaka S, Inoue K, Miyamoto K, Nakano Y, Ogawa Y, Yamada W, Shiraishi Y, Hasegawa N, Fujishima S (2008). Role of interleukin-6 in bleomycin-induced lung inflammatory changes in mice. Am J Respir Cell Mol Biol.

[9] O'Reilly S, Ciechomska M, Cant R, van Laar JM (2014). Interleukin-6 (IL-6) trans signaling drives a STAT3-dependent pathway that leads to hyperactive transforming growth factor-beta (TGF-beta) signaling promoting SMAD3 activation and fibrosis via gremlin protein. J Biol Chem.

[10] Wang JH, Zhao L, Pan X, Chen NN, Chen J, Gong QL, Su F, Yan J, Zhang Y (2016). Zhang SH hypoxia-stimulated cardiac fibroblast production of IL-6 promotes myocardial fibrosis via the TGF-beta1 signaling pathway. Lab Investig.

[11] Sheppard D (2006). Transforming growth factor beta: a central modulator of pulmonary and airway inflammation and fibrosis. Proc Am Thorac Soc.

[12] Gauldie J, Bonniaud P, Sime P, Ask K, Kolb M (2007). TGF-beta, Smad3 and the process of progressive fibrosis. Biochem Soc Trans.

[13] Hinz B (2012). Mechanical aspects of lung fibrosis: a spotlight on the myofibroblast. Proc Am Thorac Soc.

[14] Robinson MB, Deshpande DA, Chou J, Cui W, Smith S, Langefeld C, Hastie AT, Bleecker ER, Hawkins GA (2015). IL-6 trans-signaling increases expression of airways disease genes in airway smooth muscle. Am J Physiol Lung Cell Mol Physiol.

[15] Garbers Christoph, Rose-John Stefan (2018). Dissecting Interleukin-6 Classic- and Trans-Signaling in Inflammation and Cancer. Methods in Molecular Biology.

[16] Piairo P, Moura RS, Nogueira-Silva C, Correia-Pinto J (2011). The apelinergic system in the developing lung: expression and signaling. Peptides.

[17] Sakkas LI (2016). Spotlight on tocilizumab and its potential in the treatment of systemic sclerosis. Drug Des Devel Ther.

[18] Scheller J, Ohnesorge N, Rose-John S (2006). Interleukin-6 trans-signalling in chronic inflammation and cancer. Scand J Immunol.

[19] O'Donoghue RJ, Knight DA, Richards CD, Prele CM, Lau HL, Jarnicki AG, Jones J, Bozinovski S, Vlahos R, Thiem S (2012). Genetic partitioning of interleukin-6 signalling in mice dissociates Stat3 from Smad3-mediated lung fibrosis. EMBO Mol Med.

[20] Le TT, Karmouty-Quintana H, Melicoff E, Le TT, Weng T, Chen NY, Pedroza M, Zhou Y, Davies J, Philip K (2014). Blockade of IL-6 trans signaling attenuates pulmonary fibrosis. J Immunol.

[21] Yokota S, Imagawa T, Mori M, Miyamae T, Aihara Y, Takei S, Iwata N, Umebayashi H, Murata T, Miyoshi M (2008). Efficacy and safety of tocilizumab in patients with systemic-onset juvenile idiopathic arthritis: a randomised, double-blind, placebo-controlled, withdrawal phase III trial. Lancet.

[22] Khanna D, Denton CP, Jahreis A, van Laar JM, Frech TM, Anderson ME, Baron M, Chung L, Fierlbeck G, Lakshminarayanan S (2016). Safety and efficacy of subcutaneous tocilizumab in adults with systemic sclerosis (faSScinate): a phase 2, randomised, controlled trial. Lancet.

[23] Khanna D, Denton CP, Lin CJF, van Laar JM, Frech TM, Anderson ME, Baron M, Chung L, Fierlbeck G, Lakshminarayanan S (2018). Safety and efficacy of subcutaneous tocilizumab in systemic sclerosis: results from the open-label period of a phase II randomised controlled trial (faSScinate). Ann Rheum Dis.

[24] Distler Oliver, Highland Kristin B., Gahlemann Martina, Azuma Arata, Fischer Aryeh, Mayes Maureen D., Raghu Ganesh, Sauter Wiebke, Girard Mannaig, Alves Margarida, Clerisme-Beaty Emmanuelle, Stowasser Susanne, Tetzlaff Kay, Kuwana Masataka, Maher Toby M. (2019). Nintedanib for Systemic Sclerosis–Associated Interstitial Lung Disease. New England Journal of Medicine.

[25] Epstein Shochet G, Brook E, Israeli-Shani L, Edelstein E (2017). Shitrit D fibroblast paracrine TNF-alpha signaling elevates integrin A5 expression in idiopathic pulmonary fibrosis (IPF). Respir Res.

[26] Epstein Shochet G, Wollin L, Shitrit D (2018). Fibroblast-matrix interplay: Nintedanib and pirfenidone modulate the effect of IPF fibroblast-conditioned matrix on normal fibroblast phenotype. Respirology.

[27] Szklarczyk D, Gable AL, Lyon D, Junge A, Wyder S, Huerta-Cepas J, Simonovic M, Doncheva NT, Morris JH, Bork P (2019). STRING v11: protein-protein association networks with increased coverage, supporting functional discovery in genome-wide experimental datasets. Nucleic Acids Res.

[28] Pietras K, Ostman A (2010). Hallmarks of cancer: interactions with the tumor stroma. Exp Cell Res.

[29] Milara J, Ballester B, Morell A, Ortiz JL, Escriva J, Fernandez E, Perez-Vizcaino F, Cogolludo A, Pastor E, Artigues E (2018). JAK2 mediates lung fibrosis, pulmonary vascular remodelling and hypertension in idiopathic pulmonary fibrosis: an experimental study. Thorax.

[30] Pedroza M, Le TT, Lewis K, Karmouty-Quintana H, George AT, Blackburn MR, Tweardy DJ, Agarwal SK, To S (2016). STAT-3 contributes to pulmonary fibrosis through epithelial injury and fibroblast-myofibroblast differentiation. FASEB J.

[31] Denton CP, Ong VH, Xu S, Chen-Harris H, Modrusan Z, Lafyatis R, Khanna D, Jahreis A, Siegel J, Sornasse T (2018). Therapeutic interleukin-6 blockade reverses transforming growth factor-beta pathway activation in dermal fibroblasts: insights from the faSScinate clinical trial in systemic sclerosis. Ann Rheum Dis.

[32] Milara J, Hernandez G, Ballester B, Morell A, Roger I, Montero P, Escriva J, Lloris JM, Molina-Molina M, Morcillo E, Cortijo J (2018). The JAK2 pathway is activated in idiopathic pulmonary fibrosis. Respir Res.

[33] Li C, Iness A, Yoon J, Grider JR, Murthy KS, Kellum JM, Kuemmerle JF (2015). Noncanonical STAT3 activation regulates excess TGF-beta1 and collagen I expression in muscle of stricturing Crohn's disease. J Immunol.

[34] Rose-John S, Scheller J, Elson G, Jones SA (2006). Interleukin-6 biology is coordinated by membrane-bound and soluble receptors: role in inflammation and cancer. J Leukoc Biol.

[35] Scheller J, Chalaris A, Schmidt-Arras D, Rose-John S (1813). The pro- and anti-inflammatory properties of the cytokine interleukin-6. Biochim Biophys Acta.

[36] Zitman‐Gal Tali, Einbinder Yael, Ohana Meital, Katzav Aviva, Kartawy Amany, Benchetrit Sydney (2019). Effect of liraglutide on the Janus kinase/signal transducer and transcription activator (JAK/STAT) pathway in diabetic kidney disease in db / db mice and in cultured endothelial cells. Journal of Diabetes.

[37] Levy DE, Darnell JE (2002). Stats: transcriptional control and biological impact. Nat Rev Mol Cell Biol.

[38] Mohr A, Chatain N, Domoszlai T, Rinis N, Sommerauer M, Vogt M, Muller-Newen G (2012). Dynamics and non-canonical aspects of JAK/STAT signalling. Eur J Cell Biol.

[39] Sehgal PB (2008). Paradigm shifts in the cell biology of STAT signaling. Semin Cell Dev Biol.

[40] Ogata H, Chinen T, Yoshida T, Kinjyo I, Takaesu G, Shiraishi H, Iida M, Kobayashi T, Yoshimura A (2006). Loss of SOCS3 in the liver promotes fibrosis by enhancing STAT3-mediated TGF-beta1 production. Oncogene.

[41] Dees C, Tomcik M, Palumbo-Zerr K, Distler A, Beyer C, Lang V, Horn A, Zerr P, Zwerina J, Gelse K (2012). JAK-2 as a novel mediator of the profibrotic effects of transforming growth factor beta in systemic sclerosis. Arthritis Rheum.

[42] Fernandez IE, Eickelberg O (2012). The impact of TGF-beta on lung fibrosis: from targeting to biomarkers. Proc Am Thorac Soc.

[43] Koli K, Myllarniemi M, Vuorinen K, Salmenkivi K, Ryynanen MJ, Kinnula VL, Keski-Oja J (2006). Bone morphogenetic protein-4 inhibitor gremlin is overexpressed in idiopathic pulmonary fibrosis. Am J Pathol.

[44] Myllarniemi M, Lindholm P, Ryynanen MJ, Kliment CR, Salmenkivi K, Keski-Oja J, Kinnula VL, Oury TD, Koli K (2008). Gremlin-mediated decrease in bone morphogenetic protein signaling promotes pulmonary fibrosis. Am J Respir Crit Care Med.

[45] McDonough JE, Ahangari F, Li Q, Jain S, Verleden SE, Herazo-Maya J, Vukmirovic M, DeIuliis G, Tzouvelekis A, Tanabe N, et al. Transcriptional regulatory model of fibrosis progression in the human lung. JCI Insight. 2019;4. 10.1172/jci.insight.131597 PMC6948862 31600171

[46] Sato K, Tsuchiya M, Saldanha J, Koishihara Y, Ohsugi Y, Kishimoto T, Bendig MM (1993). Reshaping a human antibody to inhibit the interleukin 6-dependent tumor cell growth. Cancer Res.

[47] Alvarez D, Cardenes N, Sellares J, Bueno M, Corey C, Hanumanthu VS, Peng Y, D'Cunha H, Sembrat J, Nouraie M (2017). IPF lung fibroblasts have a senescent phenotype. Am J Physiol Lung Cell Mol Physiol.

[48] Raghu G, Chen YY, Rusch V, Rabinovitch PS (1988). Differential proliferation of fibroblasts cultured from normal and fibrotic human lungs. Am Rev Respir Dis.

[49] Baglole CJ, Reddy SY, Pollock SJ, Feldon SE, Sime PJ, Smith TJ, Phipps RP (2005). Isolation and phenotypic characterization of lung fibroblasts. Methods Mol Med.

